# Consistent spectro-spatial features of human ECoG successfully decode naturalistic behavioral states

**DOI:** 10.3389/fnhum.2024.1388267

**Published:** 2024-05-30

**Authors:** Abdulwahab Alasfour, Vikash Gilja

**Affiliations:** ^1^Department of Electrical Engineering, College of Engineering and Petroleum, Kuwait University, Kuwait City, Kuwait; ^2^Department of Electrical and Computer Engineering, University of California, San Diego, CA, United States

**Keywords:** brain-computer interfaces, neural decoding, neural signal processing, naturalistic behavior, ECoG

## Abstract

**Objective:**

Understanding the neural correlates of naturalistic behavior is critical for extending and confirming the results obtained from trial-based experiments and designing generalizable brain-computer interfaces that can operate outside laboratory environments. In this study, we aimed to pinpoint consistent spectro-spatial features of neural activity in humans that can discriminate between naturalistic behavioral states.

**Approach:**

We analyzed data from five participants using electrocorticography (ECoG) with broad spatial coverage. Spontaneous and naturalistic behaviors such as “Talking” and “Watching TV” were labeled from manually annotated videos. Linear discriminant analysis (LDA) was used to classify the two behavioral states. The parameters learned from the LDA were then used to determine whether the neural signatures driving classification performance are consistent across the participants.

**Main results:**

Spectro-spatial feature values were consistently discriminative between the two labeled behavioral states across participants. Mainly, *θ*, *α*, and low and high *γ* in the postcentral gyrus, precentral gyrus, and temporal lobe showed significant classification performance and feature consistency across participants. Subject-specific performance exceeded 70%. Combining neural activity from multiple cortical regions generally does not improve decoding performance, suggesting that information regarding the behavioral state is non-additive as a function of the cortical region.

**Significance:**

To the best of our knowledge, this is the first attempt to identify specific spectro-spatial neural correlates that consistently decode naturalistic and active behavioral states. The aim of this work is to serve as an initial starting point for developing brain-computer interfaces that can be generalized in a realistic setting and to further our understanding of the neural correlates of naturalistic behavior in humans.

## 1 Introduction

The development of brain-computer interfaces (BCI) has relied on a structured trial-based experimental paradigm, which results in specialized algorithms developed to decode specific actions predetermined by the experimenter. In a typical trial-based experimental design, behavioral complexity and variability are minimized to remove noise sources within or outside the brain and maximize the signal-to-noise ratio. Although this methodology has proven useful for BCI research and development, it suffers from multiple drawbacks inherent to task-based experiments. The neural activity generated in task-based experiments is not necessarily equivalent to the same behavior in a naturalistic setting. Additionally, the datasets are limited in size due to the nature of the experiment, which constrains the range and variability of the behaviors that the experimenter can collect.

Improved recording modalities that can simultaneously collect data from thousands of neurons and more efficient data storage methods have enabled researchers to collect data at a much larger scale (Allen et al., 2019; Stringer et al., 2019; Peterson et al., 2022). Studying naturalistic behaviors is also essential for understanding the brain outside the laboratory, where there is a need to either confirm the results obtained from experimental paradigms or amend them to accommodate ecological neural signatures.

Generalizable BCIs that can operate under a vast array of brain states and can pinpoint which behavior the participants want to perform must first be able to decode the general behavioral or internal state of the participant.

BCIs can decode a multitude of activities and behaviors. Motor control has been studied extensively (Miller et al., 2007, 2014). Algorithms have been developed to accurately translate hand movements and gestures, finger flexion (Aoki et al., 1999; Zanos et al., 2008; Foodeh et al., 2021; Mirfathollahi et al., 2022, 2023), and walking (Tortora et al., 2020). Speech prostheses have also been developed to decode word and sentence representations directly from the temporal cortex (Kellis et al., 2010; Bouchard et al., 2013; Angrick et al., 2019; Anumanchipalli et al., 2019; Moses et al., 2021; Willett et al., 2023). Visual and spatial attention-based BCIs have also been developed to enhance motor-based BCIs (Ahmadi et al., 2020; Nazari et al., 2021). Furthermore, BCI-based experiments can also be used for functional brain mapping. Along with externally measured behaviors, internal states can also be correlated with neural activity, such as thirst, learning, decision making, and autonomic tone (Peters et al., 2014; Williams et al., 2018; Allen et al., 2019; Calhoun et al., 2019). With the increase in the spatial and temporal resolution of modern intracranial neural implants, the developed algorithms have become increasingly accurate in decoding specific behaviors.

An ecological and naturalistic approach to study neural activity is a necessary step forward in BCI and systems neuroscience research (Huk et al., 2018). Motor movements spontaneously generated by participants in the epilepsy motoring unit were successfully decoded using intracranial EEG (iEEG) (Wang et al., 2016; Huk et al., 2018; Gabriel et al., 2019; Peterson et al., 2021a,b). A previous study found that naturalistic motor movements have identical neural signatures, contralateral increased high *γ* (70–110 Hz) and decreased *β* (8–32 Hz) activity, as found from traditional experiments (Peterson et al., 2021a,b). Coarsely labeled naturalistic behaviors collected over days, such as “Watching TV,” “Talking,” or “Using electronics” have also been decoded using high *γ* band and *β* band activity (Alasfour et al., 2019), albeit with a limited number of participants. It has also been found that spatiotemporal activity can discriminate between the neural activity generated from naturalistic behaviors (Alasfour et al., 2022). Naturalistic behaviors can be used to achieve transfer learning between participants, furthering the validity of an ecological approach (Peterson et al., 2021a,b). In addition to active naturalistic behaviors being decodable, internal naturalistic states, such as mood and autonomic state have been shown to be decodable with neural activity recorded from intracranial EEG (Sani et al., 2018; Alasfour et al., 2021; Bijanzadeh et al., 2022). The spectro-spatial components of different affective behaviors are similar to results obtained from the task-based method, mainly showing mood-selective activations of low frequency and high *γ* clusters across the limbic area (Sani et al., 2018). There has also been advanced in developing hardware platforms to facilitate a more ecological approach in systems neuroscience and BCI design, where a wearable platform has been developed to record intracranial neural activity as well as behavioral markers for freely moving human subjects (Topalovic et al., 2023).

This work aims to analyze the spectro-spatial components of active naturalistic behavior. Previous studies have shown that active spontaneous behaviors can be predicted from neural activity (Alasfour et al., 2019), and that multiple signal features can be used for prediction (Alasfour et al., 2022). Being able to correctly identify the behavioral state of a participant will help push future BCIs toward becoming scalable and generalizable. A generalizable BCI should work well across multiple behavioral states automatically, where it would sub-select which decoding algorithm is necessary for the given state or adjust the algorithm’s parameters accordingly. The concept of behavioral context switching has been investigated previously (Alasfour et al., 2019). A more complex and specific speech generation algorithm can be employed once a broadly behavioral state is detected, such as the “Talking.” Understanding the neurophysiological spectro-spatial features that separate these states can also guide future BCI researchers in choosing an appropriate implant location and feature extraction method.

In this work, we used the AJILE12 dataset, which consists of 12 participants in the epilepsy monitoring unit that were implanted with ECoG electrodes for clinical purposes (Peterson et al., 2022). The epilepsy monitoring unit is a specialized medical facility within a hospital that is dedicated to treating patients with drug-resistant epilepsy. Patients are implanted with intracranial EEG and monitored until a seizure occurs to pinpoint the diseased areas of the brain. In this work, a video camera recorded the patients’ activities throughout their stay. Data were collected across multiple days, and naturalistic behaviors were manually annotated from synchronized videos captured at the EMU without any experimental procedures. We mainly investigated the neural correlates separating two active states: “Talking and “Watching TV.” The following steps demonstrate that we can find spectro-spatial features that are discriminative between the two behavioral states and are consistent across participants.

## 2 Methods

### 2.1 Data preprocessing

**Figure 1:**
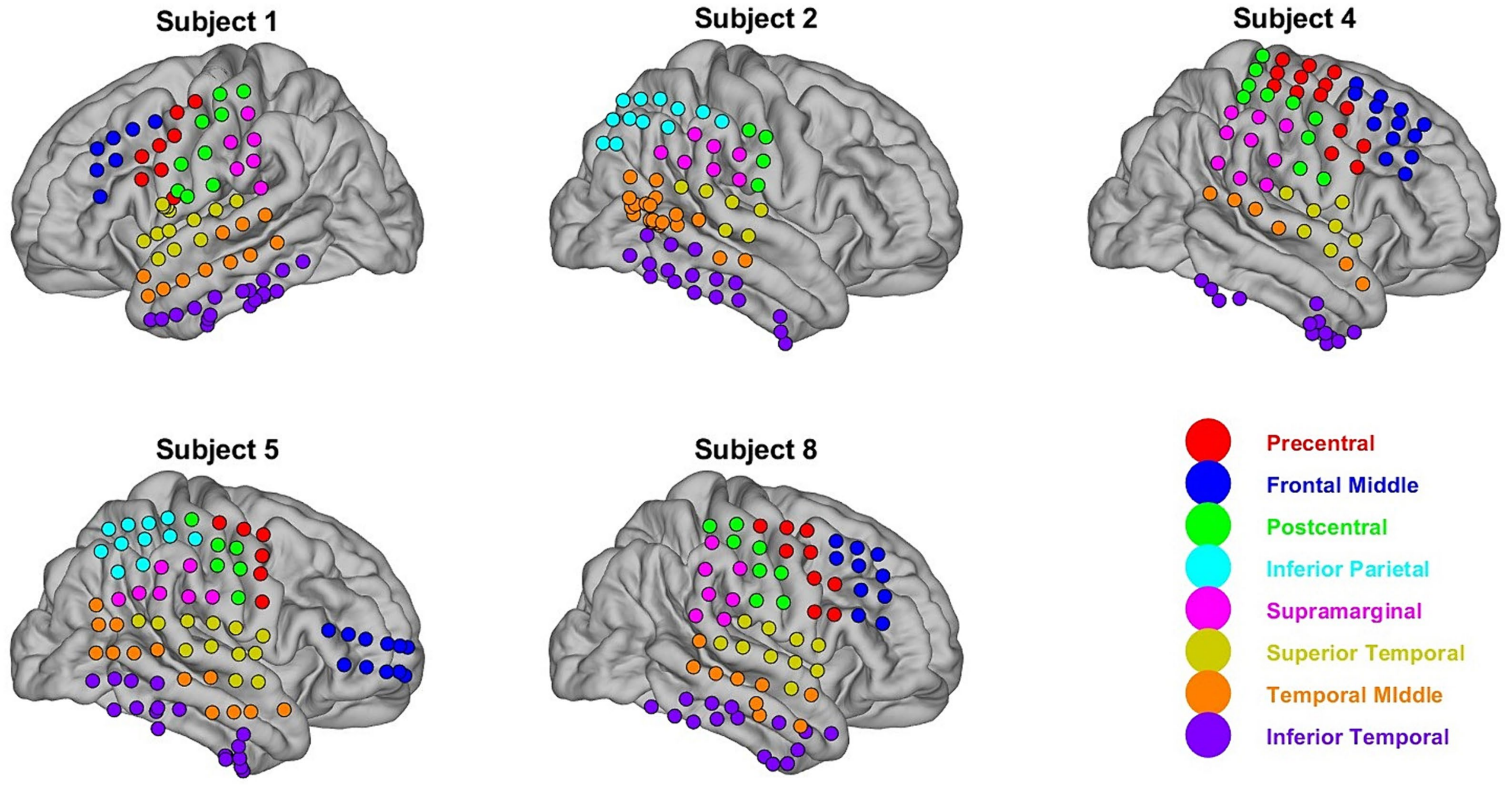
ECoG electrode positions. Eight ROI were chosen for this study using the AAL cortical atlas. Electrode positions were mapped to these regions using electrode-to-ROI projection matrices as described in Peterson et al. (2021a,b). Each ECoG electrode is color-coded according to what ROI it is mapped to.

Neurophysiological recordings were obtained from the publicly available AJILE12 dataset (Peterson et al., 2022). Five participants (2 males, 3 females) with electrocorticography (ECoG) electrodes planted on the cortical surface based on clinical needs were used in this study. We selected only participants with at least 1 h of recorded neural activity of the behaviors of interest to obtain adequate samples when training and testing our classification models. Of the 12 participants in the dataset, only 5 satisfied this criterion. Recordings lasted for an average of four days across participants and only post-surgical days 3–7 were included. The video was recorded synchronously with neural activity. The ECoG activity was recorded at a sampling rate of 1 kHz, and the video was recorded at 30 fps. The video for each participant was split into 2-min blocks, and each block was manually annotated using a coarse behavioral label. The active behavioral labels included talking, watching TV, and using a computer or phone, whereas the inactive behavioral labels included sleeping and awake rest. For most analyses in this study, we used two active labels: talking and watching TV. Naturally, when constrained to a hospital bed, the subjects’ natural tendency is to mostly engage in these two behaviors. These two labels, therefore, have a sufficient number of samples, and the analysis of the spatial-spectral features that separate them becomes tractable. Information regarding the five participants, including the number of electrodes used and the amount of data, is summarized in Table 1. Figure 1 shows the locations of the ECoG electrodes on the cortical surfaces of the five participants used in this study. More information regarding subjects not used in this study is found in the AJILE12 dataset (Peterson et al., 2022).

**Table 1:** Participant specific information.

Participant	Sex	Age	Good electrodes	Minutes talking	Minutes watching TV	Minutes using electronics
1	M	44	85	493	605	291
2	M	20	80	331	338	68
4	F	19	67	696	312	181
5	F	31	104	340	480	212
8	F	33	82	216	156	29

All five participants have at least 2 h of data for two behavioral states suitable for subsequent analysis.

Standard ECoG data preprocessing methods were used for the dataset. DC drift was removed by subtracting the median voltage and data discontinuities. The data were then bandpass filtered between 1 and 200 Hz and notch filtered at 60 Hz and its harmonics to remove line noise. Furthermore, the data were downsampled to 500 Hz and referenced to a common median across the electrode grid (Peterson et al., 2022). The ECoG channels were then passed through five IIR Butterworth bandpass filters associated with the frequency bands of interest. The five frequency bands are *θ* (4–8 Hz), *α* (8–12 Hz), *β* (8–32 Hz), low *γ* (32–55 Hz), and high *γ* (70–110 Hz). The Hilbert transform was then applied to each filtered signal to extract the envelope signal, which served as an instantaneous estimate of the frequency band amplitude. The envelope signals associated with each frequency band were then averaged across 10-s bins. Previous studies have used similar temporal resolutions to decode natural behavior and mood (Sani et al., 2018; Alasfour et al., 2019) and model the dynamic structure of multispectral long-term ECoG data (Yang et al., 2019). An artifact-rejection criterion was used to remove high amplitude bursts of activity most likely unrelated to neural activations. Any two-minute segment where 10% of channels exceeding 3 times the standard deviation of the channel across more than 2 s was eliminated from further processing.

The spatial locations of the electrodes across the cortical surface were determined using the electrode-to-region of interest (ROI) projection matrices used in a previous study that analyzed the AJILE12 dataset from the same authors (Peterson et al., 2021a,b, 2022). Electrode positions were mapped to common regions of interest in the automated automatic labeling (AAL) atlas by calculating the value of a three-dimensional Gaussian centered at each electrode position at a centroid located at each ROI (Peterson et al., 2021a,b). Each participant had a projection matrix, where each index in the matrix assessed the distance of electrode i to a region of interest j. Only electrodes local to eight defined regions of interest corresponding to cortical areas analyzed by Peterson et al. (2021a,b, 2022) were used. The matrix was discarded if the ROI had an electrode density of less than 3 for a specific participant. The projection matrices were thresholded only to include electrodes close to any of the eight defined ROIs, and the ROI label was assigned by choosing the ROI closest to each electrode. Figure 1 shows the mapping of the ECoG electrodes to the 8 defined ROIs.

### 2.2 Spectro-spatial classification and consistency analysis

Once the data had been spatially localized and frequency bands of interest were extracted, we applied a linear discriminant analysis (LDA) classifier to determine whether specific spectro-spatial features separate two coarsely labeled behaviors: “Talking” vs. “Watching TV.” The LDA classifier was applied to each ROI and frequency band for each participant. A 7-fold cross-validation scheme was applied to the ECoG activity. The data were temporally sequenced, and a one-fold buffer was applied between the training and test sets to avoid temporal correlation effects. Temporal correlation effects could influence decoding accuracy, as decodability could be due to the brain’s state at a specific period of time, rather than a function of behavior (Alasfour et al., 2019; Yang et al., 2019). The training and testing sets in each fold were downsampled to achieve a balanced amount of data between the two classes to avoid bias due to sample imbalance. Additionally, the training set and test sets were z-scored independently as a normalization step to ensure that classification is due to the neural activity difference between the two classes and to prevent slow varying trending effects influencing our results (Alasfour et al., 2019). *Z*-scoring would also aid comparisons of power differences when comparing across frequency bands since power in intracranial neural activity is concentrated in the lower frequency bands. The covariance matrices fitted to the training data were set to be equal across all classes to avoid overfitting the data to the training set and simplify the post-hoc analyses of the learned parameters. Once the classification accuracies were generated for each participant, ROI, and frequency band combination, we pooled all the participant classification accuracies to arrive at a participant-wide performance for each ROI and frequency band combination. Participant-wide accuracy was compared to chance performance while correcting for finite data, as described by Combrisson and Jerbi (2015), using a two-sided Student’s *t*-test. Significant results were determined using the Benjamini-Hochberg multiple comparison correction to control the false discovery rate (Benjamini and Hochberg, 1995). The FDR correction procedure was implemented as follows: for a total of N hypotheses, each with corresponding *p* values *Pi* (I ⊑ N), which are sorted in ascending order to identify *Pk* (*k* being the largest *i* for which *Pi* ≤ (i/N) * *α*), all hypotheses with *p* values less than or equal to *Pk* would be rejected.

An ANOVA test was also performed to determine whether there were group-level effects of the ROI, specific frequency band, and their interaction on the classification performance. To ensure that a single participant’s decoding performance did not dominate the measurements, we generated and inspected participant-specific matrices in which the rows were ROIs, the columns were the frequency bands, and the.

value was the participant-specific classification accuracy. If one participant drove the subject-wide decoding results, only that participant’s matrix would have values where the decoding accuracy was significantly greater than chance. There were sufficient data for four of the five participants to consider a third-class label, adding “Using Electronics” as an additional label. The decoding accuracy was determined for each of the five 5 frequency bands using all available electrodes from the eight 8 ROIs as features.

Decodability and classification performance alone does not give us a complete understanding of the neurophysiological features that are driving performance. In order to pinpoint how the neural activity was leading to above chance classification, we leveraged the tractability of the LDA classifier, and investigated the learned features. Because we used an LDA classifier with equal covariance matrices across classes, the only feature that drove the decoding performance is the difference between the class-specific means. For all the models trained on each fold, we extracted the fitted means for the two classes and calculated their difference. As five participants were analyzed using a 7-fold cross-validation scheme, we obtained 35 data points. This would provide a clear understanding of whether the classification is due to Class A having a higher or lower mean than Class B. The mean difference values were compared to zero using a two-sided Student’s *t*-test, and a multiple comparison correction was applied as described above. Finally, we pinpointed the ROI/frequency band combinations that drive classification performance and are consistent across patients in what drives their separability by labeling them as significant if they pass the Student’s *t*-test for both the classification performance and mean difference analysis described above. For both analyses, a Student’s *t*-test was applied to each spectro-spatial feature to determine whether the classifier performance exceeded the finite chance level or if the mean difference in the training LDA parameters differed from zero. We used FDR correction for each statistical test with *α* = 0.01 (Benjamini and Hochberg, 1995). We also investigated the consistency of the difference of neural activity between the two classes across participants. Similarly, to when investigating the consistency of classification accuracy, we generated participant-specific matrices for the differences in neural activity.

Finally, to assess whether the information within the ROIs and frequency bands was additive, we ranked the classification performance of each ROI for each participant separately. Subsequently, we assessed the performance of the classifier by starting with the highest-performing ROI and iteratively adding additional ROIs. This was performed using a 7-fold cross-validation scheme with both validation and test sets. This analysis was performed for each of the five frequency bands for each participant. This analysis aimed to determine whether additional information from other ROIs would increase the performance of the classifier, indicating whether the relevant neural activity was additive across the cortex.

## 3 Results

### 3.1 Classification accuracy and consistency of spectro-spatial features

**Figure 2:**
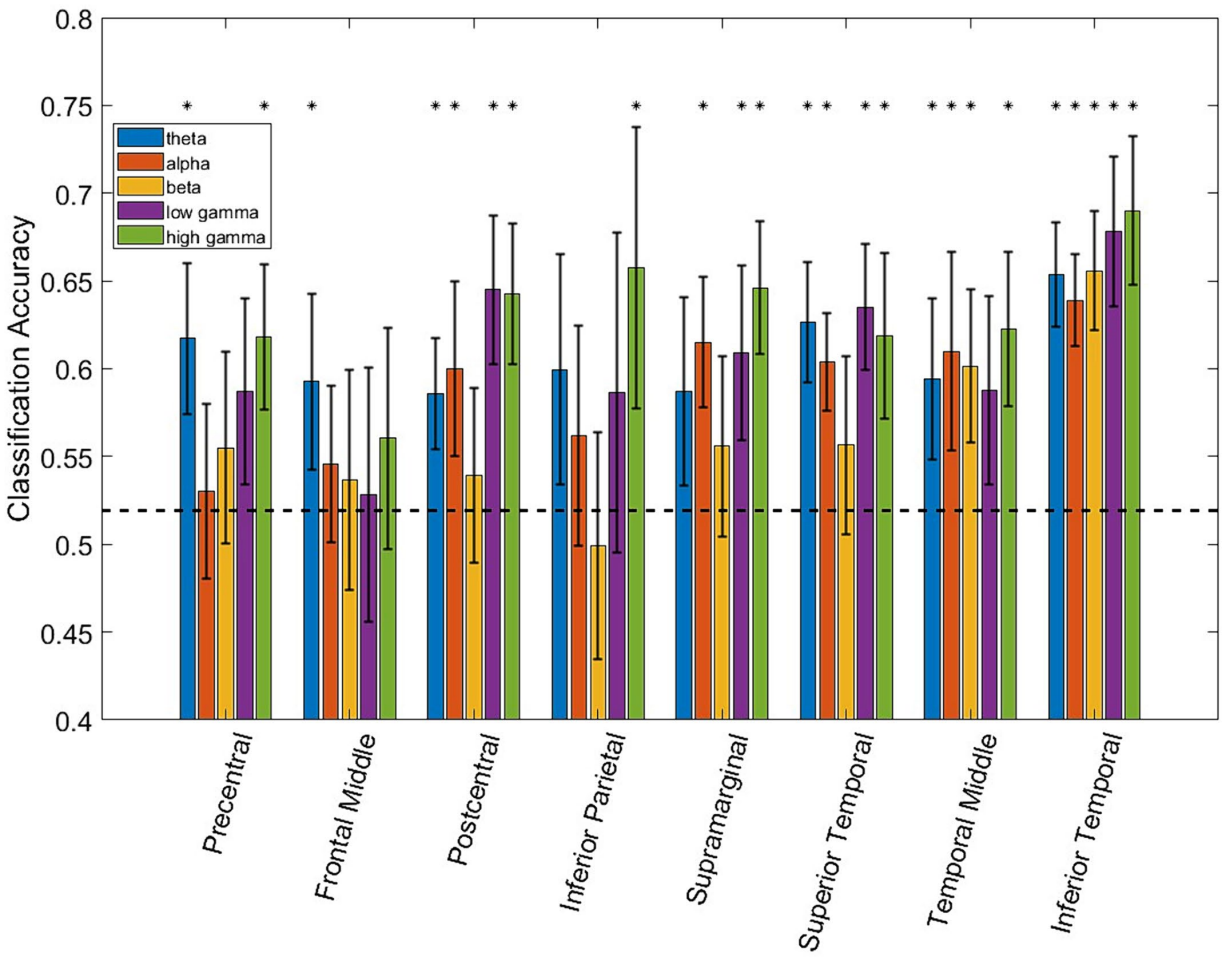
Bar plot showing the classification accuracy of each ROI/frequency band combination using LDA. The dashed black line represents finite chance performance. The black asterisk denotes performance that is statistically above chance using the Student’s *t*-test and accounting for multiple comparison testing (*p ≤* 0.01). Error bars denote the 95% confidence interval.

**Figure 3:**
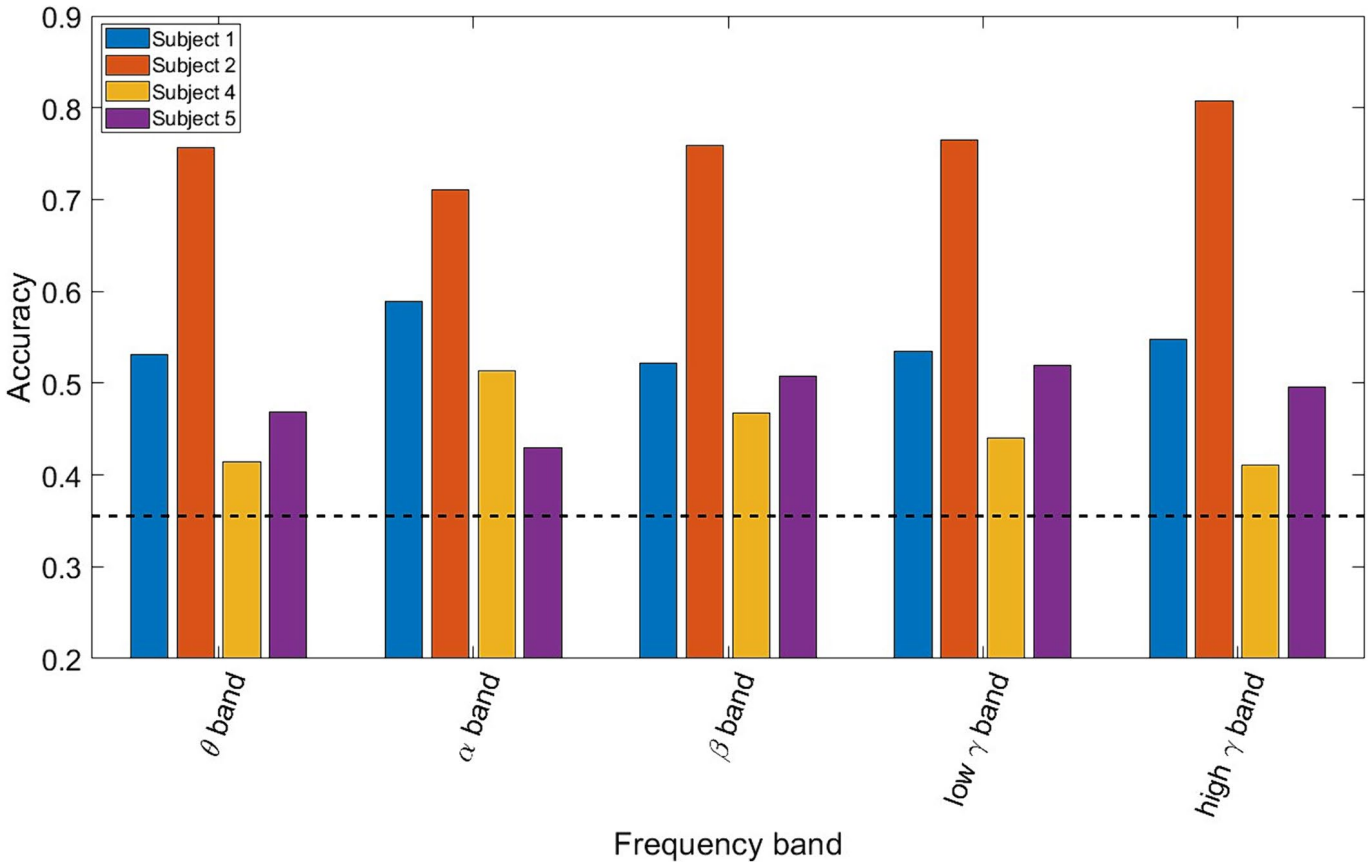
Bar plot showing the three-class classification accuracy for each participant and frequency bands using all of the electrodes. The dotted black line represents the finite chance level. All 4 participants exhibit above chance classification accuracies, with Participant 2 having the highest performance of more than 70% for all frequency bands.

Figure 2 shows the LDA classifier performance for each spectro-spatial feature (ROI and frequency band combination, for a total of 40 features). The number of data points for each element of the bar graph consists of the number of training folds multiplied by the number of participants, for a total of 35 data points. Looking at Figure 2, we observe that the highest classification performance is 69% for the high *γ* band in the inferior temporal lobe. The postcentral gyrus, supramarginal gyrus, and superior, middle, and inferior temporal lobes have 3 or more frequency bands where the LDA classification performance is significantly above chance. All 5 frequency bands in the inferior temporal lobe resulted in a decoding accuracy that was significantly higher than by chance. Both the middle temporal and inferior temporal lobe are the only ROIs where the *β* band performs above chance level. The inferior parietal and frontal middle lobes only perform above chance when high *γ* or *θ* bands are used, respectively. We also applied a two-way ANOVA test to determine whether the ROI and frequency band had a group-level effect on the classification accuracy. Both the ROI and frequency band contributed significantly to decodability, with a *p*-value of 0 for both variables. The interaction between the ROI and frequency band does not show a significant effect with a p-value of 0.46. In a post-hoc analysis of the group-level contribution to performance, we note that the high *γ* band has the highest classification performance across all ROIs, with an average of 63%. The *β* band, across all ROIs, performs the worst with an average classification accuracy of 56%, which is barely above the finite chance level. In a group-level analysis of ROIs, the inferior temporal lobe has the highest average classification accuracy across all frequency bands at 66%, and is statistically higher than all the other ROIs. The lowest performing ROI being the frontal middle lobe, with an average decoding performance of 54%. This shows that the specificity of spectro-temporal activity in iEEG recordings is essential for discriminating between naturalistic behavioral states. Finally, to verify that coarse behavioral decoding is valid in a multiclass classification paradigm, the three-class decoding performance, when adding “Using Electronics,” as a third class label is shown in Figure 3. Note that for all 4 participants, the decoding accuracy was above the finite chance level. However, there was high variability between participants. For example, Participant 2 had a much higher decoding performance than Participant 4.

**Figure 4:**
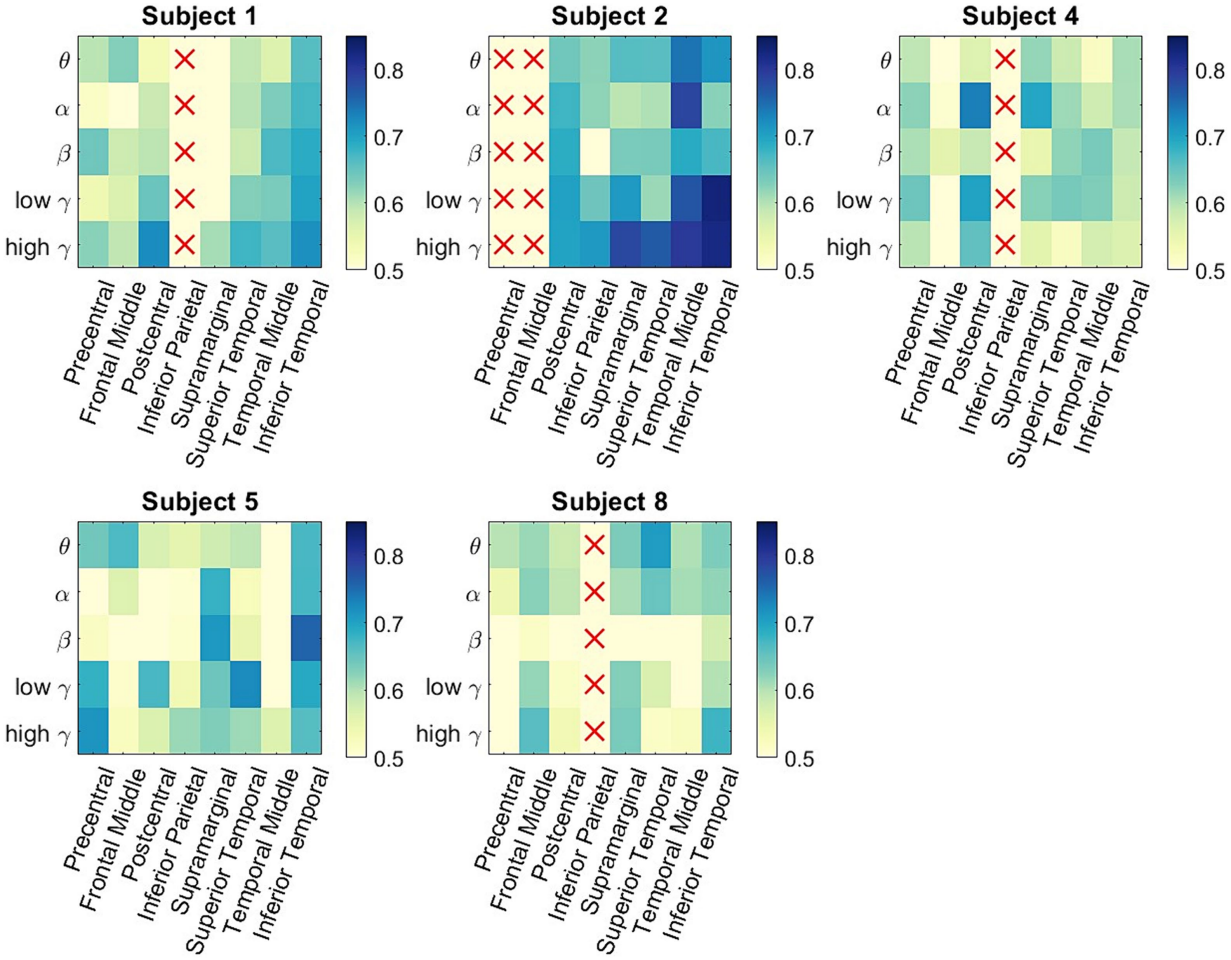
Participant-specific classification matrices. For each matrix, the rows represent the 5 different frequency bands of interest, and the columns represent the 8 studied ROIs. The color represents classification accuracy of a specific spectro-spatial feature. We observe that participant specific decoding accuracy is above chance for multiple spectro-spatial features for all subjects; thus, the group-level classification performance seen in Figure 2 is not driven by a single participant.

Figure 4 shows the subject-specific classification accuracies for each ROI and the frequency band combinations displayed as matrices. One of the issues in examining subject-wide classification scores and making neurophysiological inferences is that one participant could be the main driver of group-level performance that is statistically greater than chance. In Figure 4, we observe that all the participants have classification accuracies that are significantly above chance for multiple ROI/frequency band combinations. Note that for Participants 2 and 3, columns displayed a red x marker because no electrodes were present in that ROI. Subject 2 was the highest-performing participant, where multiple ROI/frequency band features had a classification accuracy exceeding 75%. Most notably, for Participant 2, low-and high gamma bands in the inferior temporal lobe exceeded 80% decoding accuracy.

**Figure 5:**
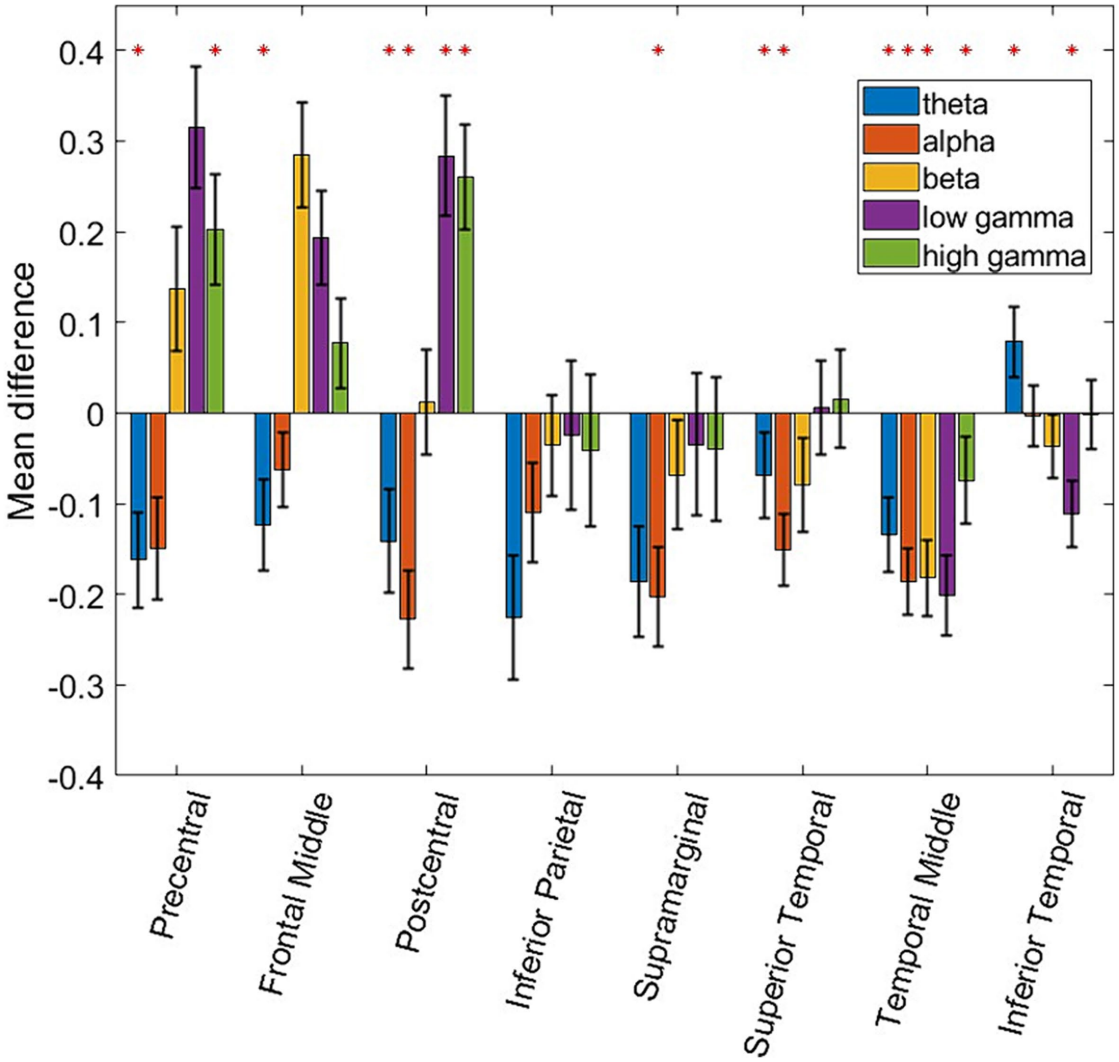
Bar plot showing the differences of fitted means in the LDA classifier. The red asterisk denotes ROI and frequency band combinations where the difference of the mean fitted by the LDA classifier for “Talking” vs. “Watching TV” is statistically different from zero. A two-sided Student *t*-test was used along with multiple comparison testing (*p ≤* 0.01). Additionally, ROI and frequency band combinations are labeled as significant if their classification accuracies are statistically above chance as seen in Figure 2. Error bars denote the 95% confidence interval.

**Figure 6:**
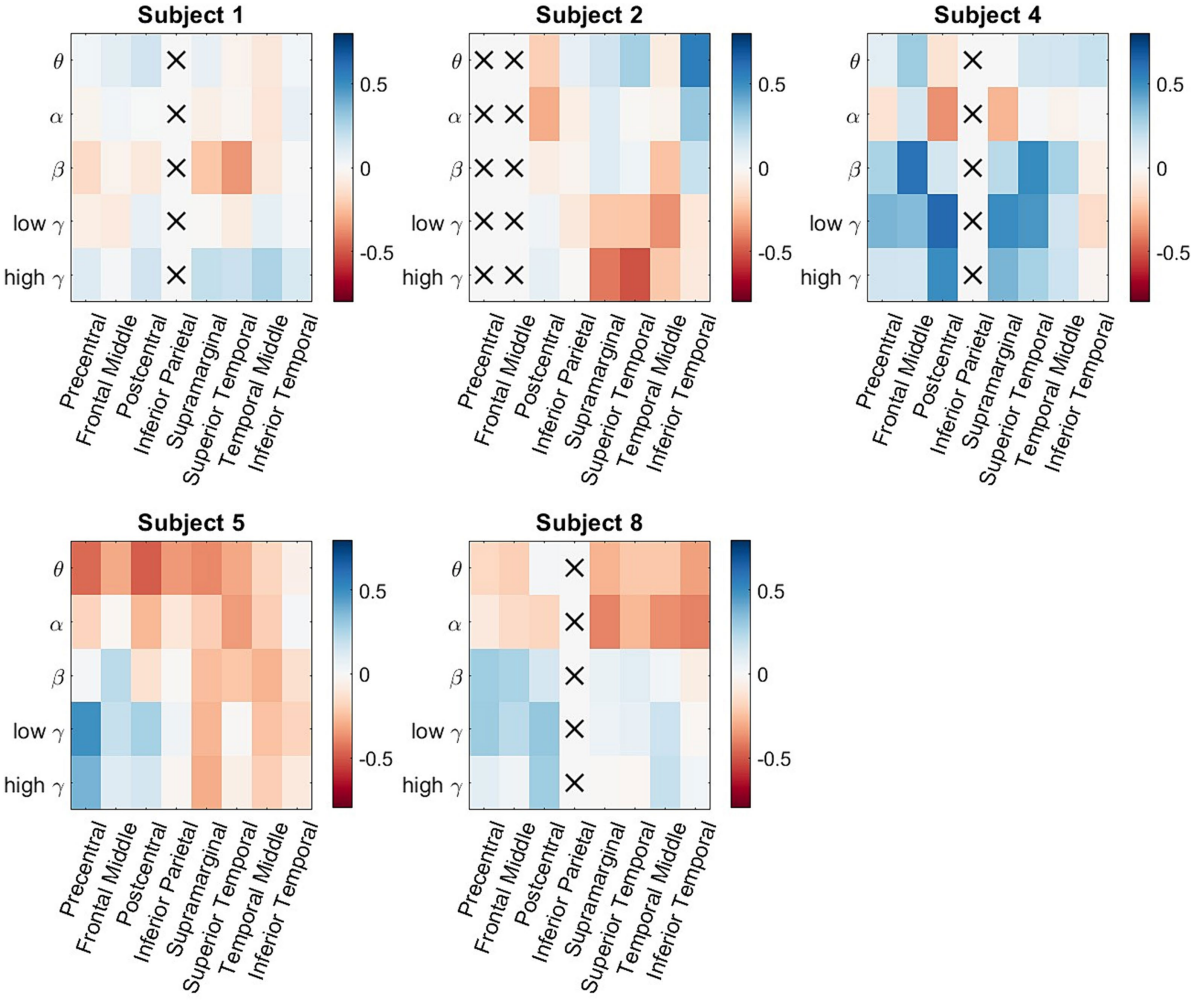
Participant-specific neural activity difference matrices. For each matrix, the rows represent the 5 different frequency bands of interest, and the columns represent the 8 studied ROIs. The color represents difference of neural activity of a specific spectro-spatial feature between the two classes. The sign of the neural activity difference of 9 out of 40 ROI/frequency band combination (22.5%) does not change across participants This result supplements our analysis of Figure 5 since the differences in neural activity seen are not driven by a single participant.

In order to generalize the classification results shown in Figures 2, 4, consistent spectro-spatial features across participants must be found. Therefore, both decodability and consistency must be present to determine the neurophysiological significance of each spectro-spatial feature. Even if a feature is decodable, this does not mean that its discriminability is consistent across different folds. For example, in one cross-validation fold, the decodability between two classes could be because the mean of class a is greater than that of class b, whereas in another fold, it is the opposite. Therefore, for this analysis, we only considered spectro-spatial features that rejected the null hypothesis using the Student’s *t*-test for both the classification performance and mean difference analyses. Figure 5 shows that consistent neurophysiological characteristics exist in the iEEG signals that specify the behavioral context in which a participant is engaged. The y-axis displays the difference in the fitted LDA classifier means between the two classes. A positive number indicates that the mean activity for “Talking” is greater than “Watching TV” and vice versa. On a macro level, we note that the low and high *γ* band is greater when a participant is engaged in talking versus watching TV. Further, *θ* and *α* band activity are lower when a participant is talking vs. watching TV. The greatest difference can be seen in the precentral and postcentral lobes, with consistent differences seen in *θ*, *α*, low *γ*, and high *γ*. When a participant engages in dialog, we do not see higher activation in the *γ* band in the supramarginal gyrus, inferior parietal and superior temporal lobe. Rather, the increased activity on *θ* and *α* is still present in these ROIs. Additionally, the middle temporal lobe displayed consistent increased activation in 4 out of 5 frequency bands of interest when the participants watched TV instead of talking. Figure 6 shows the participant-specific differences in neural activity when comparing the two classes. Each element in the matrix is the average across the LDA parameters in each training fold. A total of 9 out of 40 (22.5%) of ROI/frequency band combinations show no change in sign differences across participants, highlighting the consistency of the learned features. For example, we note that high gamma activity in the precentral, frontal middle and post central lobes all show the same directionality across the participants. In addition, alpha band is completely consistent across participants in the precentral, inferior parietal and supramarginal lobes. One must also note that the results generated in Figure 5 is the average across all training folds and across all participants, while the results in Figure 6 is the average for each participant, therefore, subject specific differences would be expected since a neural activity difference in a single training fold could heavily influence the averaged value. Finally, our results in Figure 6 show that the difference in neural activity seen in Figure 5 is not driven by a single subject.

### 3.2 Additive performance of ROI on classification

**Figure 7:**
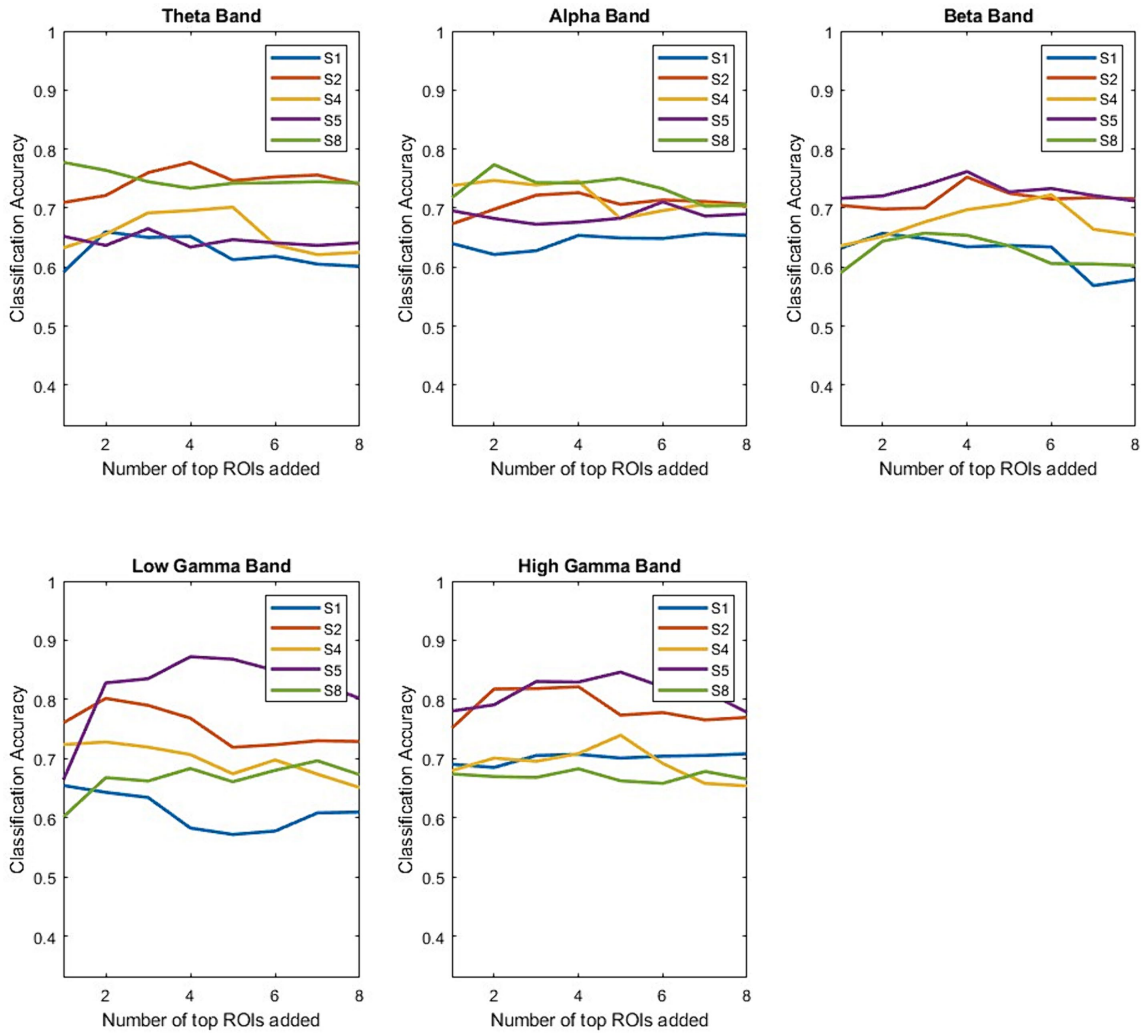
Performance of the LDA classifier after incrementally adding more ROIs. The top-performing ROIs for each frequency band are determined using a validation set. We independently computed the LDA classifier’s performance starting from the top-performing ROI for each frequency band and participant. We note that generally, performance does not increase as a function of added ROIs.

Once the decoding performance has been shown to be ROI and frequency band dependent across participants, the next step is to see if performance is additive when combining different spectro-spatial features. Figure 7 shows the participant-specific performance for each frequency band as we continued to add electrodes from the top-performing ROIs for each participant. The main trend is that the performance is not additive, indicating that adding the top-performing ROIs does not contribute additional information that would improve the decoding performance. The only instance with notable additive information is for Participant 4 when analyzing the low *γ* band. Further, we observed that for individual participants, performance usually exceeded group-level performance (Figure 2). Using a simple classifier such as LDA, we would achieve a performance of greater than 70% for all participants, with maximum classification accuracy being around 80% for Participant 4 when analyzing low and high *γ* bands. Additionally, subject-specific variability exists in the spectro-spatial features that provide the highest performance. For example, Figure 7 shows that Participant 5’s decoding performance is highest when using the *θ* band, while Participant 3’s decoding performance is highest when using the *α* band.

## 4 Discussion

This study shows specific spectro-spatial features that can discriminate between two naturalistic behavioral states: “Talking” and “Watching TV.” Even in an unstructured setting, where a participant is not instructed to perform any specific task, we can pinpoint the frequency band activity across different cortical locations that can differentiate between naturalistic behaviors. Neural activity generated from behaviors studied outside the classical task-based experimental paradigm is generally noisy, since they can be influenced by a multitude of factors such as internal states (Peters et al., 2014; Williams et al., 2018; Allen et al., 2019; Calhoun et al., 2019) and time of day (Alasfour et al., 2019). In addition, the behavior annotation is manually labeled across 2-min segments, which results in noisy labels as well. Therefore, the first step in moving away from the task-based lab protocol and toward an ecological approach to neuroscience is to determine whether naturalistic activity is separable using only neural activity, as has been shown in previous studies (Alasfour et al., 2019; Gabriel et al., 2019; Peterson et al., 2021a,b). The coarsely labeled naturalistic behaviors that we studied here have been shown to be consistent, indicating that the separability is due to the properties of the signal preserved across the training folds and participants. For example, in Figure 5, we can see that, in the precentral and postcentral lobes, *θ* band activity is generally greater while a participant is watching TV, than when engaging in dialog. The high and low *γ* bands show the opposite effect, as their activity is greater when engaging in dialog than when watching TV. Engaging in dialog involves a participant moving their mouth and/or hands, and since high-*γ* band activity can be used as a proxy for local spiking activity (Mukamel et al., 2005; Manning et al., 2009; Ray and Maunsell, 2011; Miller et al., 2014), this indicates higher activation in the motor and premotor cortices. Moving away from the motor and premotor areas, decodability was consistent across multiple frequency bands in the superior, middle, and inferior temporal lobes. The inferior temporal lobe in particular, contributed to the highest classification accuracy of around 70% for high gamma band, as well as above significantly above chance performance for all other frequency bands. It has been shown in previous work that the temporal lobe is spatially segregated when it comes to the different aspects of speech, such as listening, production, and articulation (Rampinini et al., 2017). Therefore, specific neural circuitry in the inferior temporal cortex could be responsible for ability to discriminate actively engaging in dialog as opposed to passively watching TV. It would lead us to conclude that with in this study, all analyzed ROIs, except the inferior parietal lobe, had at least one frequency band that could separate the two naturalistic behaviors and consistent signal characteristics across participants. A previous study showed that cortex-wide activity is correlated with spontaneous behaviors in mice (Stringer et al., 2019). Our results confirm that separability can be achieved across neural activity that spans multiple regions of the brain and across all frequency bands. Our results also indicate that information in this cortex-wide activity is nonadditive when it comes to decoding naturalistic behavior. Our results show that adding the top-performing ROIs generally did not improve the performance of the LDA classifier. Our results suggest that detecting coarsely labeled behavioral states depends on synchronous cortex-wide activation, as information is likely shared between different ROIs as a function of the behavioral state.

Subject-specific classification performance regularly exceeds 80% when using a simple LDA classifier. We selected an LDA classifier for this purpose because the interpretability of the parameters is straightforward. In an LDA classifier, where the covariance matrices are set to be equal across classes, the only learned parameter is the fitted means. Performance can most likely be increased by using more complex models and deep learning methods (Pandarinath et al., 2018). Not only do our results infer important neurophysiological conclusions, but they would also help guide future BCIs to become generalizable in a naturalistic setting. Current BCIs focus on decoding a specific task, such as moving an arm or talking. However, to integrate multiple decoding algorithms and operate under multiple scenarios, one must first detect the behavioral state of the participant.

A behavioral context switch (Alasfour et al., 2019) can be implemented using the results obtained in this study to switch between different decoding algorithms, depending on the behavioral context. Some practical applications include when a context switch detects that a person is watching TV, it can implement one of the many studied cursor-movement decoding algorithms so that the participant can switch channels. A speech-decoding algorithm can be applied if the context switch detects that the participant is engaging in dialog. Additionally, detecting idle states is vital so that the BCI is not activated when the participant is relaxing or asleep, thus preserving battery life.

Our analysis was based on manually annotated coarse behavioral states. Although we achieved above chance decodability for all the participants analyzed, this is not an optimal labeling strategy for training models implemented in practical BCIs. Significant advances have been achieved in the detection of patient poses using naturalistic videos (Chen et al., 2018; Peterson et al., 2021a,b) for concurrent analyses of neural signals. These techniques help track movement, which would be ideal for creating models correlating naturalistic movements to neural activity. However, advanced computer vision techniques are required to segment videos and identify different behavioral states semantically. This could aid not only in the automation of labeling, which would lead to more accessible data collection, but also in identifying exactly when a participant starts or ends a specific behavior. The temporal resolution in this dataset is in the order of minutes, but with the ability to increase the temporal resolution, one can precisely pinpoint the neural correlates that identify a behavioral switch. A more detailed analysis of neural data can be achieved with a computer vision approach that would increase the temporal resolution.

Recently, there has been a boom in the application of deep learning methods for decoding neural data, such as spiking (Pandarinath et al., 2018) and LFP signals (Angrick et al., 2019; Makin et al., 2020). Deep learning networks are usually seen as black boxes representing nonlinearities, and the presence of multiple layers in the deep learning model makes the interpretability nontrivial. However, recent advances have been made in interpreting the parameters to derive neurophysiological conclusions (Peterson et al., 2021a,b; Stuart et al., 2022) and, in our work, we applied simple linear models to pinpoint exactly why we can decode coarsely labeled behavioral states. When using LDA with equal covariance, the only parameter that is learned is the mean value of the fitted Gaussians. Therefore, we can determine why we can separate the two classes. Although a finely tuned deep learning model would outperform our decoding performance in this study, we aimed to serve two primary functions in the neuroengineering pipeline. First, it can aid future researchers in the feature engineering step, as understanding which spatiospectral features are discriminatory is critical in any neuroengineering task. Second, we determined that multiple regions of the cortex spatially provide information regarding naturalistic behavioral states; therefore, strategies for implant design need to consider the cortex-wide involvement of naturalistic behavior. Our results indicate that recording neural activity from multiple ROIs can marginally improve performance for a subset of patients, and that the relationship between decoding performance and the amount of coverage is not straightforward. Our results raise additional important questions regarding implant design. When investigating high γ activity in the inferior temporal lobe, the decoding performance was generally high, whereas the mean difference was virtually zero. This means that although this ROI and frequency band combination is highly informative, the neural signal behavior is inconsistent across cross-validation folds and participants. This could motivate the use of a higher-density electrode coverage on specific ROIs to determine whether intra-participant signal consistency and decodability can be achieved.

The next step in tackling naturalistic behaviors is to determine whether one can recover a dynamic structure in the data. A hallmark finding in neuroengineering is the presence of latent dynamics within neural activity, which are mainly observed in motor-related tasks (Pandarinath et al., 2018). Previous studies have shown that discriminable spatiotemporal dynamics exist in naturalistic behavior (Alasfour et al., 2022) and that a dynamical model can be fitted to ECoG data recorded in an unconstrained setting (Yang et al., 2019). Therefore, a natural extension of this work is to determine whether a latent dynamic structure drives naturalistic behavior to gain insight into the neurophysiological underpinnings of naturalistic behavior. Understanding the latent structure of naturalistic neural activities can drive the algorithmic design of generalizable BCIs.

## 5 Conclusion

The trial-based experimental protocol has been the gold standard for elucidating the neurophysiological correlates of behavior and driving the algorithmic design of BCIs. In this study, we confirmed the ability to decode coarsely labeled naturalistic behaviors in a larger set of participants using ECoG from a previous study. Spectro-spatial features obtained from ECoG neural activity collected from multiple cortical regions are shown to successfully separate two behavioral states: “Talking” and “Watching TV.” We used a linear covariance LDA model to find these features and show that they both contain information that decodes behavioral states and are consistent in their signal characteristics across 5 participants. Although multiple ROIs can be used to decode naturalistic behavior independently, the information contained is generally non-additive, indicating a cortex-wide brain state shift correlated with behavior. To our knowledge, this is the first attempt to elucidate the neurophysiological nature of naturalistic and spontaneous behaviors.

## Data availability statement

Data used in this study is publicly available and can be downloaded from https://dandiarchive.org/dandiset/000055 (Peterson et al., 2022).

## Ethics statement

The studies involving humans were approved by University of Washington Institutional Review Board. The studies were conducted in accordance with the local legislation and institutional requirements. The participants provided their written informed consent to participate in this study.

## Author contributions

AA: Conceptualization, Data curation, Methodology, Writing – original draft, Writing – review & editing, Formal analysis. VG: Supervision, Writing – original draft, Writing – review & editing.
